# Another one bites the dust…

**DOI:** 10.5195/jmla.2017.37

**Published:** 2017-04

**Authors:** Frank Houghton

“A Magazine Is an iPad That Does Not Work” [1]

Last week, I received one of those dreaded annual emails. That’s right, the now almost routine one asking faculty to voice their support, or lack thereof, for the ever-declining number of hard copy journals still being purchased by the library. As an associate professor of public health recently promoted to chair of the department, I appreciate their position, of course. The dominance and ease of online access to journal databases increasingly marginalizes such resources. The very idea of actually reading a hard copy of an article in its natural context (that is, within the actual physically printed issue) seems increasingly quaint, if not to say ludicrous, as time goes on. In an era of budget constraints and concerns over value for money, the library faculty and staff are only showing due diligence in financial oversight.

Not that I am against electronic access, far from it. Such developments have revolutionized our access to what seems sometimes to be an almost overwhelming volume of information. I have often tried, usually in vain, to describe to current students the complicated, cumbersome, and painfully slow process that was involved in actually identifying a single relevant journal article when I went to college almost thirty years ago. As I attempt to describe the weighty tomes involved in this now defunct process, I usually end up drawing on scenes from *Harry Potter* and the Hogwarts library to assist in my description [2].

Perhaps I am old fashioned or simply a fossil. Admittedly, at a personal level, I still prefer to read the hard copy. However, even putting this bias aside, I do wonder what else is lost as the online journal database access transformation continues. Although I am conscious of the advantages of online access, online-only access via library databases presents a number of disadvantages that should not be overlooked. Other writers have explored in-depth a number of issues associated with online access including slower speed of reading, decreased accuracy in recall, increased fatigue, lower comprehension [3], and sleep disturbance [4]. Some authors have looked at the negative aspects of online learning in relation to distraction and multitasking [5], loss of a sense of control, and how reading a particular book, *War and Peace,* for example, should “feel” [1]. Research has also suggested a reduced breadth and depth of learning in online education, as well as the almost geographical advantages of books in aiding both recall and helping readers navigate their way through and around texts [1].The list of potential negatives continues with some researchers noting the adverse impact of the use of electronic shortcuts on online learning [5, 6], while others focus on the tactile [3], kinesthetic physicality in reading [1]. It has been suggested that the most basic acts of reading such as the turning of pages and tracing of words by a finger— alongside the sounds, feel, and scents associated with the experience—deepen our understanding, memory, and engagement with the reading process.

However, valid as these commentaries may be, my own particular interest focuses on two additional areas of concern: serendipity and “complete” journals. The first of these is in part testimony to the success of online databases. This issue relates to a potential loss of breadth and richness in our learning. As search operations in our online databases become increasingly sophisticated and accurate, so we inevitably start to exclude other publications that appear less relevant. Our knowledge undoubtedly becomes more specific and focused and probably deeper as well. However, perhaps we lose breadth in our learning and with this, some of our understanding of the interconnectedness of our fields. The very act of the routine reading of a contents page and the incidental knowledge gathered in flicking through the pages of the hard copy while searching for a particular article, or just perusing for items of interest, should not be dismissed. Serendipity in learning should never be underestimated. Such “unintentional knowledge” strengthens us and feeds our appetite for new knowledge [7, 8]. Armed with an open mind and intellectual curiosity, this unintended learning is invaluable in the development of an up-to-date and broad knowledgebase.

Another potential loss in the age of online journal database access is the reduction in students’ awareness of the full range of academic products that many journals routinely include. Take for example the *American Journal of Public Health,* which invites twelve different types of submission: research articles, brief articles, systematic reviews, letters and responses, editors’ choice, opinion editorials, commentaries, analytic essays, history essays, voices, news, and images. Lack of familiarity with and understanding of the broad structure of journals and their richness and diversity is a loss. The apparent Holy Grail of publishing a peer-reviewed research article (often involving a double-blind randomized control trial) stymies intellectual debate, commentary, and connection. Learning about the diversity of publication types facilitates access to more engaging and more accessible material. At its most basic level, this broader learning can even include the artwork on the cover of the journal. As an academic who worked for almost two decades in Ireland, I learned to appreciate the often modernist artwork on the cover of the *Irish Journal of Psychological Medicine,* which challenged and changed my taste in art.

My resistance to online only access is muted. How does one quantify such intangibles? In an era of budget contraction, even I cannot articulate coherent arguments in defense of library-purchased print copies. However, I do fear the loss…

## References

[1] Jabr F (2013). The reading brain in the digital age: the science of paper versus screens. Scientific Am.

[2] Rowling JK (1997). Harry Potter and the philosopher’s stone.

[3] Dillon A (1992). Reading from paper versus screens: a critical review of the empirical literature. Ergonomics.

[4] Myrberg C, Wilberg N (2015). Screen vs. paper: what is the difference for reading and learning?. Insights.

[5] Niccoli A (2015). Paper or tablet? reading recall and comprehension. EDUCAUSE Rev.

[6] Liu Z (2005). Reading behavior in the digital environment. changes in reading behavior over the last ten years. J Documentation.

[7] Alves J (2013). Unintentional knowledge: what we find when we’re not looking. Chron Higher Educ.

[8] Berg M, Seeber BK (2016). The slow professor: challenging the culture of speed in the academy.

